# Sequencing technology in sarcopenia: current research progress and future trends

**DOI:** 10.3389/fmolb.2024.1309006

**Published:** 2024-09-03

**Authors:** Yuxia Yang, Xiangji Meng, Xiaomei Dai, Jian Zhang, Jihang Dai, Jingcheng Wang, Wenyong Fei

**Affiliations:** ^1^ Department of Orthopedics and Sports Medicine, Northern Jiangsu People’s Hospital Affiliated to Yangzhou University, Yangzhou, China; ^2^ Department of Orthopedics and Sports Medicine, Northern Jiangsu People’s Hospital Affiliated to Dalian Medical University, Dalian, China

**Keywords:** sarcopenia, muscle atrophy, gene sequencing, protein sequencing, metabolic sequencing, bibliometric analysis

## Abstract

**Background:**

Muscle is an important tissue of the human body. Muscle atrophy is common in people of all ages, which will lead to human weakness and decline of motor function, which is one of the important causes of disability. The common methods of genomics research are transcriptome, proteomics and metabolomics, which are important means to explore the molecular pathology of diseases. In recent years, combinatorial research has been carried out on a large scale in the field of muscle atrophy. However, no author in this field has carried out bibliometrics and visual analysis.

**Methods:**

In this study, articles related to the histological study of muscular dystrophy since 2000 were searched from the Web of Science core database (WoSCC). We will retrieve the results through CiteSpace, VosViewer and R for data statistics and visual analysis.

**Results:**

In this study, a total of 141 publications were collected, and the number of publications increased year by year. These 141 articles came from 1031 co-authors from 361 institutions in 31 countries and were published in 92 journals. A total of 6286 articles from 1383 journals were cited. Authors from American institutions have published the most articles and have been cited the most, and authors from other countries have also made considerable contributions.

**Conclusion:**

This is the first bibliometric and visual analysis of published research in the field of muscular dystrophy through systematic data retrieval and combined with a variety of bibliometric analysis tools. Through these data, we summarize the previous studies of scholars, and provide prospects for future research in the field.

## 1 Introduction

As the most abundant tissue of the human body, muscle plays an important role in human movement, respiration and glucose homeostasis (Exeter and Connell, 2010). However, in many cases, muscle response to specific stimuli, such as muscle waste, denervation, drugs or malignant diseases, will lead to loss of muscle mass and function, resulting in muscle atrophy (muscle aging) (Schmidt et al., 2019). Muscle atrophy is common in people of all ages, which can lead to human weakness and decline in motor function, and is one of the important causes of death in the elderly (Yin et al., 2021). According to statistics, by 2018, the prevalence rate of muscular dystrophy among 70-year-olds worldwide is about 10%, while the proportion of 80-year-olds has risen to 50% (Cruz-Jentoft et al., 2010). In young people, there are often patients with muscle atrophy due to limitation of muscle use and imbalance of muscle metabolism caused by trauma hospitalization or other diseases (Yin et al., 2021). Since the pathophysiological mechanism of muscular dystrophy has not been clearly explained, it cannot be attributed to a single factor (Schiaffino et al., 2013). At present, the main clinical treatments for muscular dystrophy are biotherapy, such as platelet concentrator, growth factor, stent, drug therapy, such as non-steroidal anti-inflammatory drugs, steroid hormones, rehabilitation therapy, such as continuous passive exercise, and hyperbaric oxygen therapy (Wang et al., 2021; Howard et al., 2020; Bao et al., 2022). However, these treatments can not effectively delay the decline of muscle mass and function, so it is necessary to find out the detailed occurrence and development mechanism of muscle atrophy from many aspects.

In recent years, due to the rapid development of science and technology, genomics and systems biology have gradually become an important means to study the molecular pathology of diseases (Liu et al., 2023; Vandereyken et al., 2023). In 2003, Welle S identified the gene expression profile of human muscle aging in the muscles of healthy young and old men (Welle et al., 2003), Piec I analyzed the differential proteome of aging skeletal muscle and healthy skeletal muscle in 2005 (Piec et al., 2005), and then the histological study began to be carried out on a large scale in the field of muscle atrophy. The common methods of genomics research are transcriptome, proteomics and metabolomics. Proteomics is the study of proteins, which can detect a large number of different proteins in tissue and provide the molecular state of tissue protein level (Ubaida-Mohien et al., 2019). Transcriptome is the sum of all RNA transcribed by a particular tissue or cell at a certain developmental stage or functional state, mainly including mRNA and non-coding RNA, which provides information on the transcriptional level of a certain state in the tissue (Rao et al., 2021). Metabonomics is based on the idea of transcriptome and proteomics to quantify the metabolites in the tissue and to find the relative relationship between metabolites and physiological or pathological changes. In addition, it also includes epigenomics, single cell biology, liposome and so on (Miao et al., 2021). The combination of these combinatorial methods can provide a comprehensive understanding of the molecular mechanisms of the disease at different levels, and provide a holistic and comprehensive point of view (Hu et al., 2018). Therefore, the joint multi-group analysis of muscle atrophy is helpful to understand the occurrence mechanism in more detail and find new targets for therapeutic intervention.

In the past 10 years, the number of studies related to muscular dystrophy has increased rapidly, but there is still a huge knowledge gap, a large number of literatures, scholars cannot understand each one. Therefore, a scientific and effective method is needed to evaluate the latest and important progress of research and summarize the new trends in the pathogenesis of muscular dystrophy. Professor Paul Otlet first defined bibliometrics as a scientific discipline in 1934 and regarded it as a research method (Rousseau, 2014). The scientific method of bibliometrics can help researchers grasp and understand the overall situation of a certain field and evaluate the published research results, which is of great significance to determine the Frontier and development direction of a certain research field (Liu et al., 2022; Liu et al., 2021). In bibliometric research, the commonly used methods include citation analysis, cluster analysis, correlation analysis and so on (Shadgan et al., 2010).

Exploring the mechanism of disease occurrence and progression is an important step in clinical-oriented transformation. In this paper, we searched the Web of Science core database (WoSCC) for articles related to the histological study of muscular dystrophy since 2000. We use the retrieved results for screening summary and visual analysis to evaluate the contributions of countries, institutions, journals and authors to specific research topics. The purpose of this paper is to analyze the current research status of muscular dystrophy in the world, comprehensively understand the latest research hotspots and development trends, and look forward to the future research direction.

## 2 Methods

### 2.1 Data sources and search strategies

WOS is the most commonly used literature retrieval database used by scholars, including a wide range of high-quality journals and comprehensive citation records. In order to ensure that the retrieved data is sufficient and comprehensive, this study retrieves and downloads the data through WoSCC’s extended Science Citation Index (SCIE) and Social Science Citation Index (SSCI). We have systematically searched the articles published from 1 January 2000 to 1 September 2023, whose types are article and review. The language is set to English, and there are no restrictions on nationality and major. The search strategy is: ((TI = transcriptomic) OR (TI = proteome) OR (TI = proteomics) OR (TI = metabolomics) OR (TI = metagenomics) OR (TI = metatranscriptomics) OR (TI = omics) OR (TI = microarray) OR (TI = RNA-seq) OR (TI = sequencing) OR (TI = ATAC-seq) OR (TI = “single cell sequencing”) OR (TI = “single-cell omics”) OR (TI = “ single cell sequence”) OR (TI = “single cell RNA sequencing”) OR (TI = “single cell RNA sequence”) OR (TI = “expression profile”) OR (TI = epigenomics) OR (TI = bioinformatic*) OR (TI = high throughput) OR (TI = mass spectrometry)) AND ((TI = muscular atrophy) OR (TI = muscle atrophy) OR (TI = sarcopenia) OR (TI = amyotrophy) OR (TI = skeletal muscle atrophy) OR (TI = muscle ageing)). We export all search results from WOS in plain text format and store them temporarily.

### 2.2 Data collection

In order to avoid the errors caused by frequent database updates, we searched and downloaded WoSCC data on 1 September 2023, and downloaded a total of 141 articles that met our screening criteria. The “bibliometrixpackage4.0.1″of R software (version4.2.1) is a tool for bibliometric analysis and data collation and collection. We import the data information into R software for integrity check and cleaning, and import key information such as title, author, year, country, institution, journal and keywords into Microsoft Office Excel (version 2019) for preservation and follow-up analysis.

### 2.3 Bibliometric analysis

The results of bibliometric analysis can be better reflected through scientific mapping and visual analysis, and this process requires the participation of several software. First of all, we counted the author, organization, country and other information through R software. Excel is an industry-leading spreadsheet software program and a powerful data visualization and analysis tool. We count the proportion of articles published, the total number of citations and the average number of articles cited each year through Excel. Citespace (version6.1. R3) is a citation visualization analysis software gradually developed under the background of scient metrics and data visualization. It focuses on analyzing the potential relationships contained in scientific literature, and can intuitively understand the research hotspots and evolution processes in various fields of the knowledge system, and foresee its development trend. VOSviewer (version1.6.18) is a visual analysis software used to construct and view bibliometric maps. Its main purpose is to analyze bibliometrics networks and create visual maps, so as to fully understand the structure and dynamic development of scientific research. We used this two software to build a visual coupling network between journals, countries, co-authors, and keywords, identify citation burst data, build visual maps, and analyze research trends and research hotspots in the field

## 3 Result

### 3.1 Publication analysis of omics studies in muscular atrophy

**FIGURE 1:**
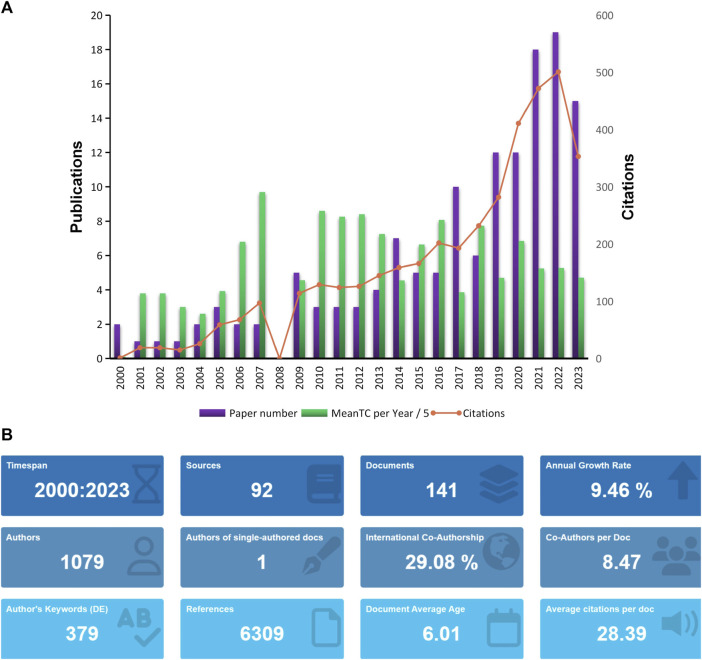
Analysis of published papers. **(A)** Year distribution and citations of omics studies in muscular atrophy. **(B)** General information about papers related to omics studies in muscular atrophy.

Through the search strategy mentioned in the methods section, we retrieved a total of 141 eligible articles (Figure 1A). The time span of these articles is from 2000 to 2023, and the number of publications increases year by year, mainly showing two stages. The first stage is from 2000 to 2020, and the number of articles published in this interval increases slowly; while the second stage is from 2020 From 2023 to 2023, the number of articles published in the past few years has increased dramatically, indicating that research related to omics in muscle atrophy has become a research hotspot in recent years. The most cited year is 2022. According to statistics from R software, these 141 articles come from 1,031 co-authors from 361 institutions in 31 countries and were published in 92 journals. These articles cited a total of 6286 documents from 1383 journals (Figure 1B).

Based on the data from WOS statistics, three of the most cited articles are *Gene expression profile of aging in human muscle* published by Welle, S in 2003 in the journal *PHYSIOLOGICAL GENOMICS*, which has been cited 251 times up to now; the second article is *Nicotinamide Riboside Augments the Aged Human Skeletal Muscle NAD (+) Metabolome and Induces Transcriptomic and Anti-inflammatory Signatures* published by Elhassan, YS in the journal *CELLREPORTS* in 2019, it has been cited 192 times; and the third article is the *Aging and microRNA expression in human skeletal muscle: a microarray and bioinformatics analysis* article published by Drummond, MJ in the journal *PHYSIOLOGICAL GENOMICS* in 2011, which has been cited 165 times.

### 3.2 Visualization analysis of keyword co-occurrence

**FIGURE 2:**
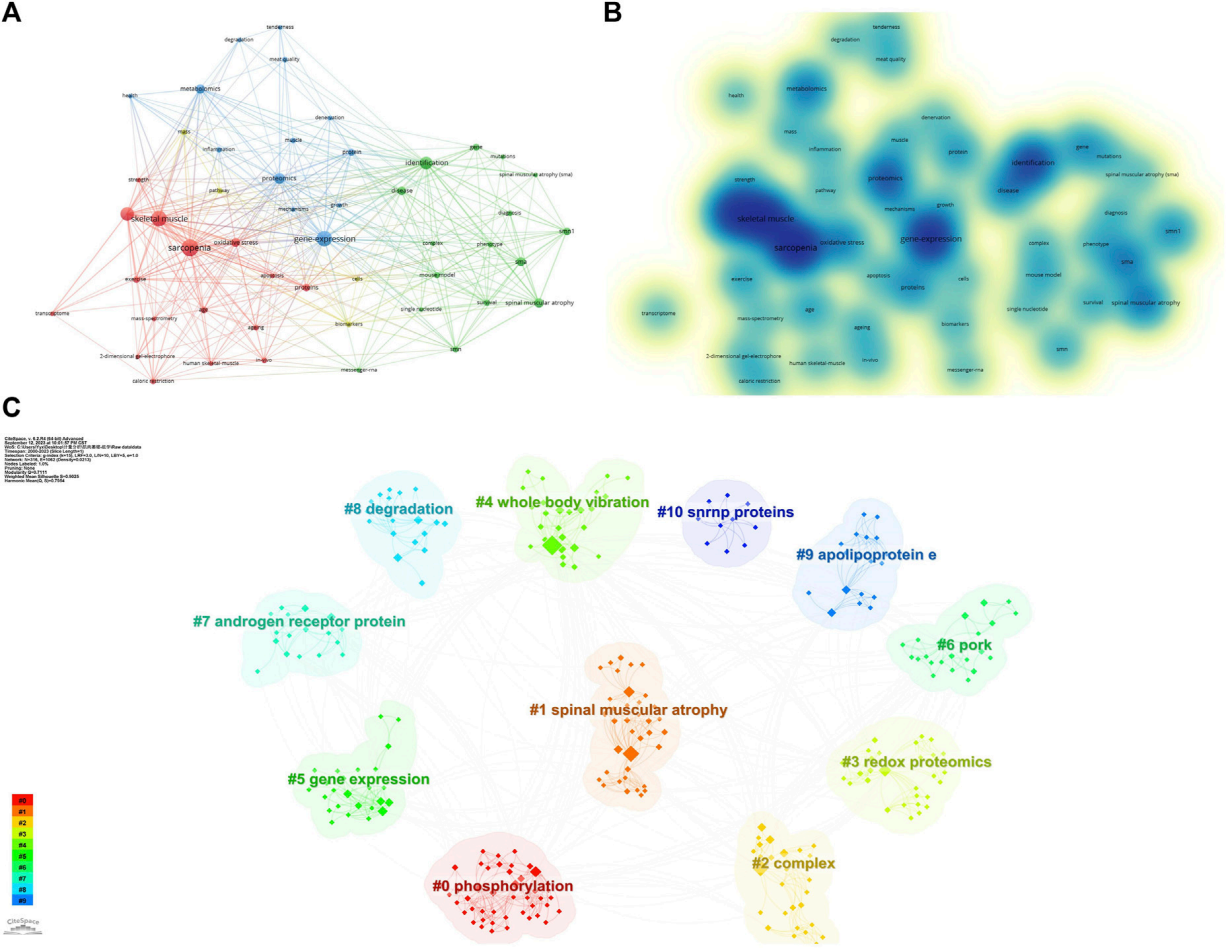
Keywords related to omics studies in muscular atrophy. **(A)** Visualization of keyword drawn by the VOSviewer. **(B)** Density visualization map of the keywords. **(C)** Keyword clustering situation, different colors represent different cluster groups.

The keywords of an article can represent its theme and main points. By constructing a recurrence map of keywords, the research hot spots and trends in the field can be described. We used VOSviewer to visually analyze keywords that appeared 5 times or more in the included articles, and obtained a total of 52 keywords. According to (Figures 2A, B), the larger the circle represented by the keyword, or the darker the color of the keyword, the higher the frequency of the keyword, and the more it represents the research hotspot in the field; in addition, the difference between the two keywords Connected lines indicate that they co-occur in the same article, and their thickness is proportional to the frequency of co-occurrence. It can be observed that the five most frequently used keywords in the field of lung cancer mitochondria research are: “sarcopenia”, “aging”, “identification”, “expression”, and “skeletal muscle”. We performed a cluster analysis of the keywords that appeared through Citespace software, and a total of 11 clusters were calculated, namely, #0phosphorylation, #1spinal muscular atrophy, #2complex, #3redox proteomics, #4whole body vibration, #5gene expression, #6pork, #7androgen receptor protein, #8degradaton, #9apolipoprotein, #10snrnp proteins (Figure 2C), different keywords were calculated to be included in different clusters.

**FIGURE 3:**
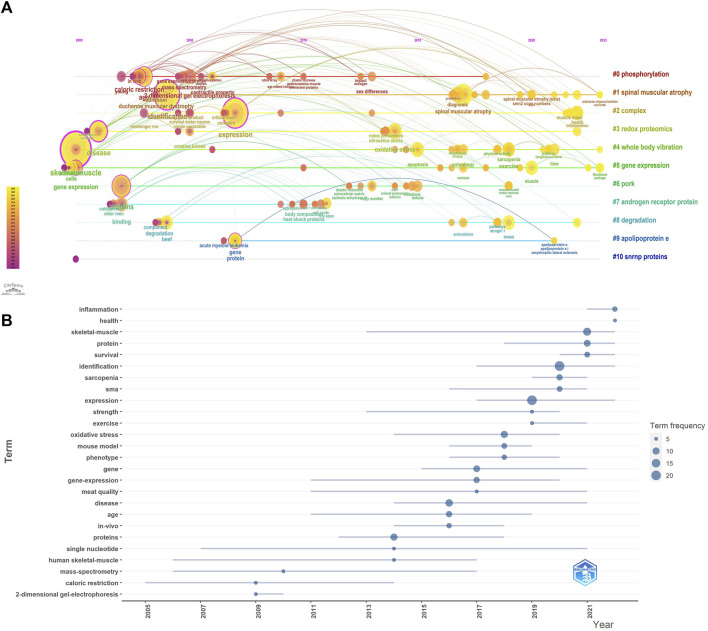
**(A)** Timeline graph of keywords related to omics studies in muscular atrophy between 2000 and 2023. Circle size represents frequency of occurrence, with red representing more recent occurrences. **(B)** Trends in research topics over time.

In order to visualize the changes of keywords over time, we constructed a keyword timeline graph (Figure 3A), where the node size represents the frequency of recent occurrences, the color represents the average time of occurrence, and the color changes from purple to yellow with the increase of the chronological order, and some keywords “fibroblasts package”, “antisense oligonucleotides accurate”, “redox proteomics”, “apoptosis” appear later, indicating a new direction of research in the field. It can be seen that the keyword evolution process within cluster #0phosphorylation is the richest and has the deepest influence on each cluster. In addition, we calculated Trends topics by R software, (Figure 3B), shows the temporal evolution process of Trends topics, the blue line represents the duration of occurrence, the size of the circle represents the frequency of occurrence, and some research topics are similar to the temporal evolution of the keywords, such as “identification”, “expression”, and “skeletal muscle”, which have been in the focus of research in this field.

### 3.3 Analysis of journals

**FIGURE 4:**
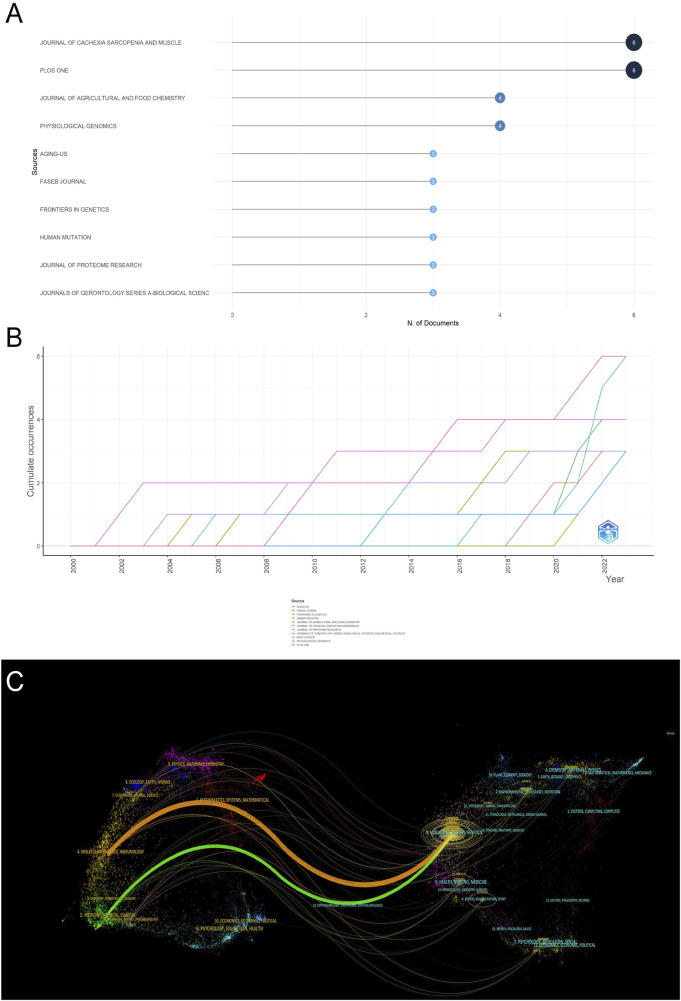
Analysis of journals. **(A)** The 10 most relevant journals participating in publishing articles. **(B)** Year distribution of the 10 most relevant journals participating in publishing articles. **(C)** Double image overlay of journals.

In the research field of muscle dystrophy research, we used the R package to calculate the 10 most relevant publishing journals (Figure 4A). *Journal of Cachexia Sarcopenia and Muscle* has the highest number of publications, with a total of 6 articles; followed by the journal *Plos one*, a total of 6 articles. As a special issue on muscle research, *Journal of Cachexia Sarcopenia and Muscle* has contributed a lot of high-quality literature. Most of the other journals participating in the publication are comprehensive journals. At the same time, we display the 10 most relevant journals in a publication time distribution chart (Figure 4B). In addition, we also used a double overlay map of journals to analyze the relationship between citing journals and cited journals (Figure 4C). The left side represents the citing journal, the right side represents the cited journal, and the colored path represents the relationship between the citing journal and the cited journal. Citation relationships between cited journals. This figure mainly shows that in muscle omics research, articles on molecular science, biology, and immunology mainly cite articles on molecular science, biology, and genetics, while articles on medicine, pharmacy, and clinical medicine mainly cite articles on molecular science, biology, and biology. Articles on science and genetics.

### 3.4 Network visualization map of references

**FIGURE 5:**
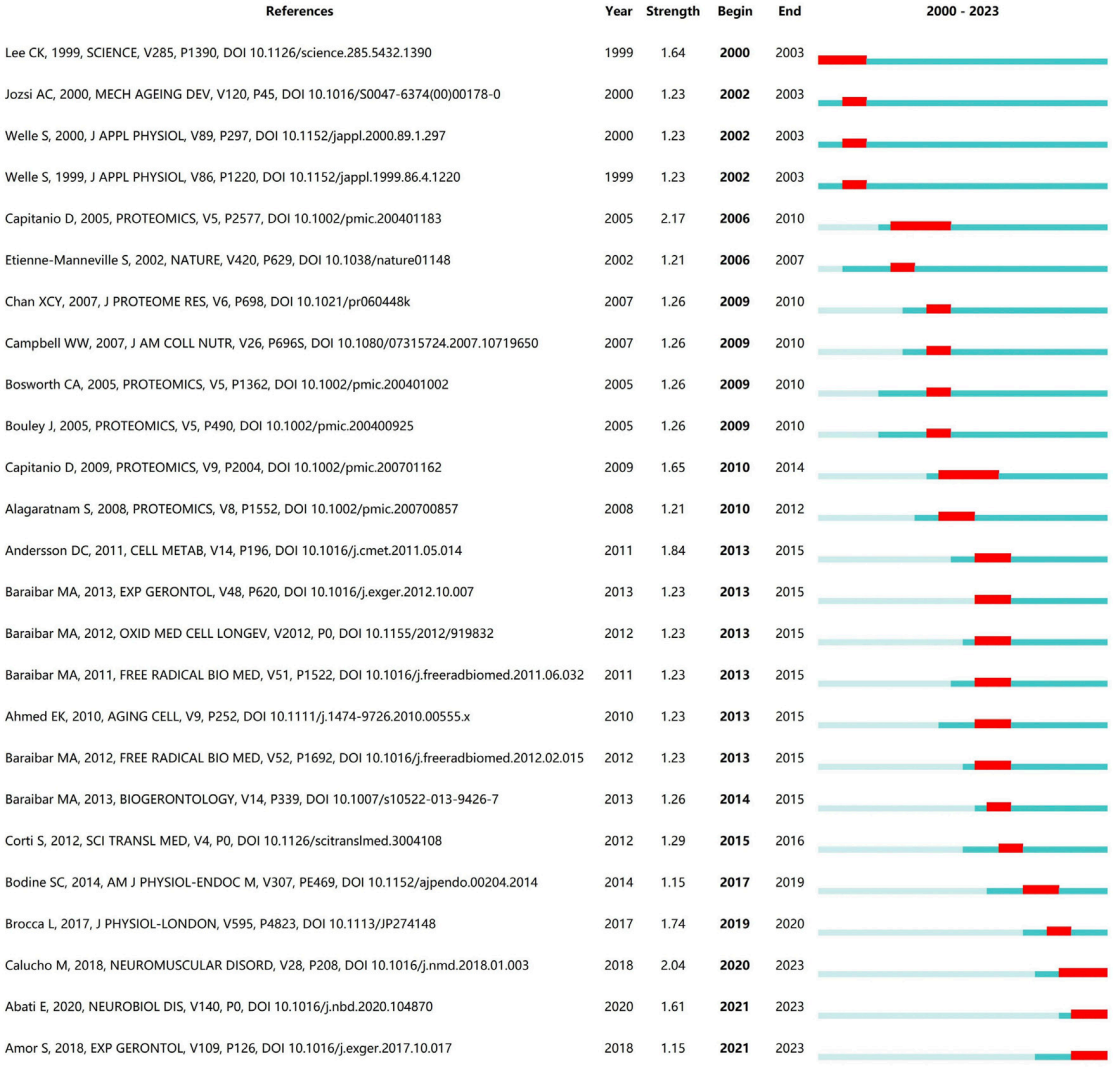
The 25 references with the highest burst intensity.

The references cited by the article can reflect the research direction of the document to a certain extent. (Figure 5). shows the 25 references with the highest burst intensity among the included articles. The length of the blue line represents the duration of the document being cited, and the red line segments represent highly referenced time periods. As seen in Figure 5, the literature with the highest intensity of outbreaks are *Comparison of protein expression in human deltoideus and vastus lateralis muscles using two-dimensional gel electrophoresis* by Daniele Capitanio’s team, published in 2005 in the journal *Proteomics*, and the *Correlation between SMA type and SMN2 copy number revisited: An analysis of 625 unrelated Spanish patients and a compilation of 2834 reported cases* by Maite Calucho’s team published in 2018 in the journal *Neuromuscul Disord*.

### 3.5 Analysis of countries

**FIGURE 6:**
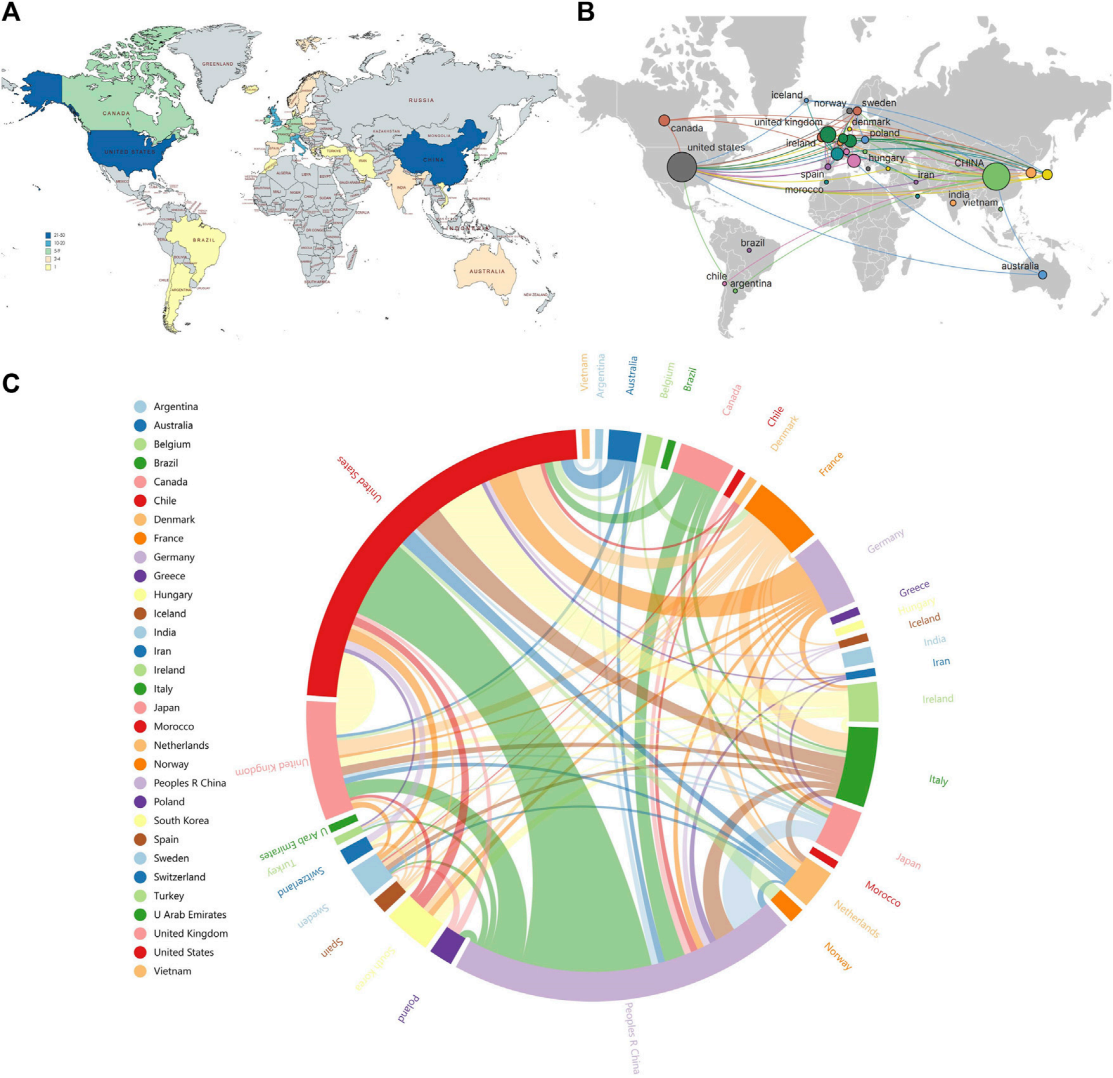
Distribution and linkage of country composition related to omics studies in muscular atrophy. **(A)** Distribution of publications by country. **(B)** Visualizing networks for international research collaboration. **(C)** Chord diagram for assessing international cooperation networks.

Among the 141 publications in the field of omics research in muscle atrophy, we conducted a visual network analysis of the countries that participated in publishing the articles. The number of statistical countries was not limited to the first author and corresponding author, but was distributed in a total of 31 countries. These countries are mainly distributed in Europe (Figure 6A). Among them, United States has the highest number of articles, reaching 51, followed by China with 44 articles and the United Kingdom with 15 articles. The countries with the highest number of citations are the United States with 2,286 citations, the United Kingdom with 659 citations, and Italy with 439 citations. (Figures 6B, C). shows the visual network of international cooperation. The size of the circle and chord diagram area represents the number of citations. It can be observed that most cooperative relationships are related to the United States, and cooperation with China is also relatively close. Most of them revolve around developed countries, while cooperative relationships between other countries are relatively weak.

### 3.6 Analysis of organizations

**FIGURE 7:**
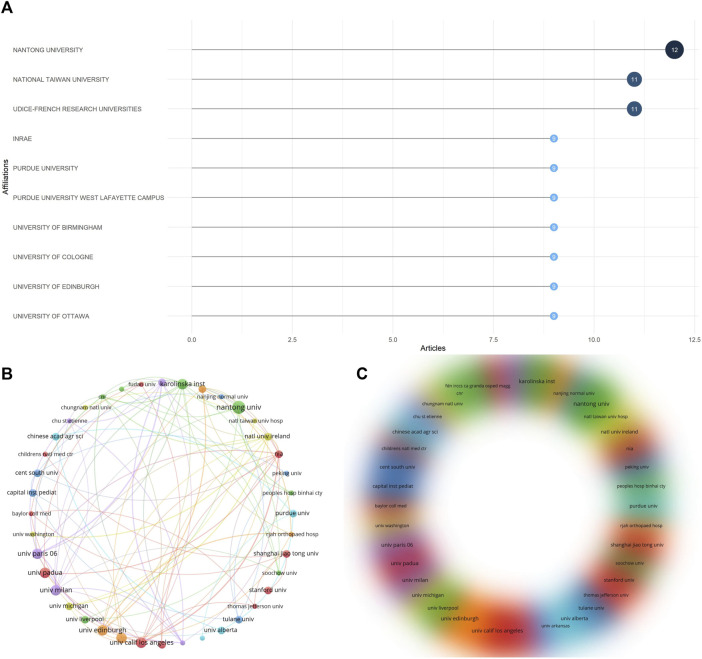
Analysis of co-authorship organizations. **(A)** The 10 most relevant institutions among publications. **(B)** Visualized contact diagram of co-authorship institutions. **(C)** Co-authorship density visualization map.

Through data statistics, a total of 361 organizations participated in publishing the article, and Figure 7A shows the 10 most relevant organizations. Among them, Nantong University participated in the most publications (12 articles); followed by National Taiwan University and Udice-French Research University, both participated in 11 publications. We used the VOSViewer software to conduct network visualization and density visualization analysis of the publishing organizations (Figures 7B, C). The lines show the cooperative relationships between various organizations. The size or density of the circles represents the number of citations. We set the minimum number of cooperations to 2 and constructed cooperative relationships between 54 organizations. Each organization cooperates more with the three institutions of Univ Edinburgh, Univ Calif Los Angeles and Karolinska Inst. The institutions related to these authors maintain a stable group cooperation research model with each other, and there is relatively little cooperation between other institutions.

### 3.7 Analysis of authors

**FIGURE 8:**
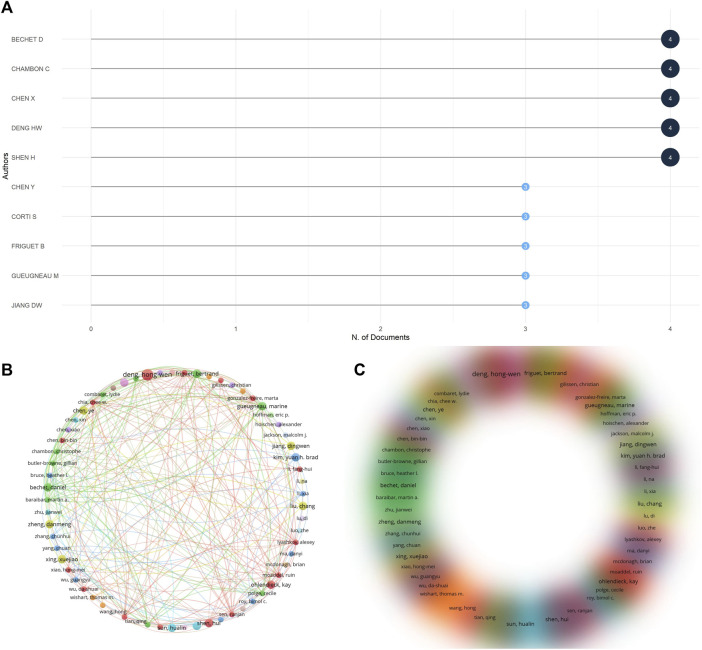
Analysis of co-authorship authors. **(A)** The 10 most relevant authors among publications. **(B)** Co-author visual contact diagram. **(C)** Co-author density visualization map.

A total of 1,031 authors were involved in contributing through data calculation, and the top 10 most relevant authors are shown in Figure 8A, mostly within the biology research field, and most of these authors were involved in publishing three to four papers, and they contributed greatly to the omics research in muscle atrophy (Figures 8B, C). Lists the co-authorship network of some closely connected authors. The minimum number of collaborations between authors is 2. A co-authorship network involving 65 authors is drawn. The lines show the collaboration between each author. Relationship, circle size or density represents the number of citations. It can be seen that the cooperation between the authors mainly revolves around authors such as Bechet D, Chambon C and Chen X. Cooperation between authors is mainly carried out in the form of groups. These authors mainly use institutions as the mode of collaborative research, and there is relatively little cross-institutional cooperation.

## 4 Discussion

Muscle is a key tissue for human movement, metabolic homeostasis and heat production. Muscle accounts for 40% of human body weight and 50% of total protein (Dolan et al., 2019). However, when some specific stimuli (chronic wasting disease, local inflammation, muscle disuse, aging) can cause an imbalance in the decomposition and homeostasis of myofibrillar protein synthesis, causing muscle atrophy (Yin et al., 2021). The pathological characteristics of muscle atrophy are a reduction in muscle mass and muscle fiber volume, and the overall performance is a sharp decline in muscle strength and function (Baehr et al., 2022). For the treatment of muscle atrophy, functional exercise is considered to have preventive and therapeutic effects. However, exercise is not suitable for all patients. Although many scholars are currently working hard to develop active drugs for the treatment of skeletal muscle atrophy, there is no recognized effective drug. Drugs are widely used (Morley and Anker, 2017; Siff et al., 2021). Therefore, it is necessary to study the key targets of muscle atrophy to provide theoretical basis for prevention and early treatment.

Previous studies have shown that skeletal muscle maintains its own homeostasis relying on the balance between protein synthesis and breakdown. Scholars attribute the imbalance of muscle protein homeostasis to the following aspects: ubiquitin-proteasome system, autophagy-lysosome system, oxidation Stress, inflammation, hormones, signal transduction (Odeh et al., 2020). In recent years, new mechanisms of action have been continuously discovered, such as insulin resistance and mitochondrial dysfunction (Kalinkovich and Livshits, 2017; Dantas et al., 2022). There are a large number of relevant research articles, so it is necessary to comprehensively evaluate the important results in this field using scientific and practical methods.

Bibliometric research originated in the field of information science. With the development of the times, computers and the Internet have gradually promoted it to become an independent scientific major and is widely used in various research fields (Aria and Cuccurullo, 2017) This bibliometric study searched as comprehensively as possible for papers on different omics studies of muscle atrophy, clustered and iterated the data through mathematical statistical methods, and conducted quantitative analysis on the number, citations, and correlations of academic results. Objectively measure and evaluate research results, quickly grasp key research hotspots and future research trends in this field, and provide references for academic research.

Our statistical publication trends indicate that research related to omics in muscle atrophy is growing rapidly year by year (Figure 1). Among them, related research has increased dramatically in the past 3 years, indicating that omics research has gradually become a research hotspot in the field of muscle atrophy in recent years and is being applied by more and more scientific researchers. As a carrier of research results, journals help disseminate important results in this field. Most of the articles included in this search were published in cell biology and biology journals such as *PHYSIOLOGICALGENOMICS* and *CELL REPORT*S; scientific researchers are also willing to publish their research in special issues on omics research such as *JOURNALOF PROTEOMERESEARCH* and *JOURNALOF PROTEOMICS*, as well as special issues on muscle research such as *JOURNAL OF CACHEXIA SARCOPENIA AND MUSCLE*. Among the articles included in this study, there are many transcriptomic, proteomic and metabolomic studies related to human muscle atrophy, while there are relatively few other omics studies. Interestingly, Ma and DY reported in 2017 on the skeleton of aged cattle (Ma et al., 2017). Metabolomic research on muscle shows that omics research has also been deeply applied in animal science. The most cited article is the research article *Gene expression profile of aging in human muscle* published by author Welle S in 2003. The author sampled skeletal muscle specimens from healthy adults and elderly men and identified transcript changes in the aging process of human skeletal muscle. The research currently has been cited 251 times (Welle et al., 2003).

Keyword analysis of articles can help scholars discover research hot spots and predict future research directions. We conducted detailed statistics on the keywords appearing in these articles and found that the keywords with the highest frequency are “sarcopenia”, “aging”, and “identification”. Due to the lack of specificity of these keywords, they are closely related to muscle atrophy and aging. Related. We analyzed other frequently occurring keywords and found that among omics-related keywords, keywords such as “gene” and “gene-expression” appear most frequently, indicating that transcriptomics-related research has been carried out the most, followed by “proteomics” and “protein”, and “metabolomics”, indicating that the number of proteomics and metabolomics studies is second to transcriptomics studies. Among the studies included in our search, there were transcriptome and proteome studies from 2000 to 2023, and metabolomics studies only gradually increased after 2015. Other genomic studies, such as single cell biology and epigenomics, had not found relevant articles until 2020, until 2022, when Junjie S carried out single-cell RNA sequencing in mice with spinal muscular atrophy, which provided a new clue for spinal muscular atrophy (Sun et al., 2022). It is worth noting that few authors have combined multiple combinatorial methods into research before 2020, and since then researchers tend to explore the mechanism of disease by combining multiple combinatorial methods (Roberts et al., 2022; Faravelli et al., 2023). Interestingly, there are many types of muscular dystrophy/muscular aging, and we found that many types of muscular dystrophy were studied in the studies included in the search, such as neuromuscular dystrophy (Ehmsen et al., 2019), disuse muscular atrophy (Cui et al., 2020), spinal muscular atrophy (Mo et al., 2010), and cachexia muscular atrophy (Blackwell et al., 2018). The application of combinatorial research provides new insights into the complexity of muscular dystrophy diseases.

International cooperation can quickly promote the progress of research. It can be clearly seen from the national distribution map that the United States is not only an important contributor to muscular dystrophy research, but also the center of international cooperation (Figures 6A–C). It can be observed that most of the cooperative relations are related to the United States, and the cooperation with China is relatively close, mostly around developed countries, while the cooperative relations between other countries are relatively weak. It shows that the international cooperation in muscular dystrophy-related research is mainly around the United States, China, the United Kingdom and other countries. We believe that there are obvious regional differences in the histological study of muscular dystrophy, and the main reason for this difference is the degree of development of national technology and scientific research. Countries with better technology and scientific research are more likely to play a leading and core role in related fields. The number of articles published does not reflect the research level of a country, so we count the number of citations of national literature. The highest countries are the United States (2286), Britain (659) and Italy (439). The United States has the highest number of articles and citations, which shows that American research in this field has been recognized by scholars all over the world.

As the United States accounts for the highest proportion of published papers, most of the institutions belonging to these articles come from the United States, and several institutions in China have also made great contributions, indicating that there are great national and regional differences in the development of genomics research. In the cooperative network of institutions, it is almost around Univ Edinburgh, Univ Calif Los Angeles and Karolinska Inst, while the links between other institutions are weak. H index is widely used to evaluate the academic output and level of researchers, and quantifies the influence of researchers as independent individuals in the field. In short, H index means that at most H papers have been cited at least H times. (Shah and Jawaid, 2023). Among the articles included in the study, many researchers have made outstanding achievements in this field, among which the Mann, Matthias scholar from Max Planck Institute of Biochemistry is an influential author. His articles have been included in the WOS database with a total of 399 articles. He has maintained a stable output of scientific research achievements in the past 20 years, mainly related to various genomics-related studies, with a total cited frequency of 26334 times and H index of 78. Thornton, Charles A from University of Rochester is also a very influential author. WOS has collected 188 articles he co-authored, mainly engaged in sports system-related research, with a total cited frequency of 13996 times and an H index of 65. In addition, there are many outstanding authors whose H index is higher than 50. These are excellent researchers in the field of interdisciplinary medicine and cell biology, mostly from well-known institutions in the United States and Europe. There is a close collaborative research model between these authors. However, it also shows that the research in this field is unevenly distributed all over the world.

As far as we know, this study has some limitations. First, we retrieved and downloaded the data only through the WOS database. Although we have repeatedly adjusted the retrieval format, it is inevitable that we cannot find some articles that cannot be retrieved. Secondly, the articles that have been published for a long time have been cited by more scholars, and later authors are also willing to quote articles with high citation rates in the past, so the number of citations cannot fully reflect the content and quality of the articles. Interestingly, we found that articles published after 2015 still appeared in front of the highest cited frequency, largely avoiding this problem. Although scholars have made a lot of efforts on the road of exploring the mechanism of muscular dystrophy, there is still a lot of room for exploration, which is believed to be a research hotspot for a long time in the future. We have reason to believe that with the progress of experimental techniques, new combinatorial studies will be more and more applied to the study of muscle atrophy, paving the way for uncovering the pathogenesis of muscle atrophy. In view of the above problems, it is necessary to continuously pay attention to the scientific research achievements related to muscular dystrophy in the next few years and keep abreast of the latest research trends in time.

## 5 Conclusion

In general, we creatively combined a variety of bibliometric tools to analyze the histological study of muscular dystrophy. It is understood that this study is the first time to make a comprehensive bibliometric evaluation of the achievements in this field. Our search papers cover most of the necessary research, and the number of research has increased sharply since 2020. The United States has been a leader in this field, and many excellent institutions and authors have made outstanding contributions. In these articles, for many types of muscle atrophy, a large number of studies on transcriptome, proteome and metabolic group have been carried out, while studies such as single-cell omics have been gradually carried out in recent years, and more attention may be paid to the study of combined multi-omics in the future

## Data Availability

The raw data supporting the conclusions of this article will be made available by the authors, without undue reservation.
